# Lymph node cancer of the mediastinum with a putative necrotic primary lesion in the lung: a case report

**DOI:** 10.1186/s12957-018-1373-y

**Published:** 2018-04-02

**Authors:** Daichi Shikata, Takahiro Nakagomi, Rumi Higuchi, Yujiro Yokoyama, Toshio Oyama, Taichiro Goto

**Affiliations:** 1 Department of General Thoracic Surgery, Yamanashi Central Hospital, Yamanashi, 400-8506 Japan; 2 Department of Pathology, Yamanashi Central Hospital, Yamanashi, Japan

**Keywords:** Mediastinal cancer, Lymph node cancer, Unknown primary site, Immunohistology, Surgery

## Abstract

**Background:**

Although mediastinal lymph node cancer is presumed to originate in the lung, the primary site is usually unidentified, so the pathological course remains unclear. We recently encountered a case of mediastinal lymph node cancer having a putative primary lesion remaining in the lung as a necrotic focus.

**Case presentation:**

The patient was a 56-year-old man who visited our department because computed tomography screening had revealed a nodular shadow in the lingular segment. However, on positron emission tomography, fluorine-18 deoxyglucose accumulation was detected in a subcarinal lymph node and not in the nodule in the lingular segment. Biopsy of the lung tumor and the lymph node was performed via minimal thoracotomy. Intraoperative pathologic examination showed necrosis alone and no malignant findings in the lung tumor. By contrast, carcinoma was detected in the lymph node. Additional subcarinal lymph node dissection was performed. Results of postoperative histopathologic examination indicated poorly differentiated adenocarcinoma of the subcarinal lymph node. Meanwhile, the nodule in the lingular segment was speculated to be a spontaneously resolved primary focus of lung cancer.

**Conclusions:**

In this case, the primary lung cancer focus resolved spontaneously after lymph node metastasis, explaining the pathogenesis underlying mediastinal lymph node cancer of unknown primary site. For similar cases of malignancy, aggressive treatment, including surgery, is effective.

## Background

Cancer of unknown primary site (CUP) rarely occurs in the mediastinal lymph node, and its underlying pathology is typically not elucidated [1, 2]. Many cases of mediastinal lymph node cancer are presumed to arise from the lung, but the primary focus remains essentially unidentified at the time of diagnosis [1, 3–5]. Although patients are usually treated by chemoradiation therapy or surgery, no standard treatment has been established. We recently encountered a case of mediastinal lymph node cancer with a putative necrotic primary lesion in the lung.

## Case presentation

**Fig. 1:**
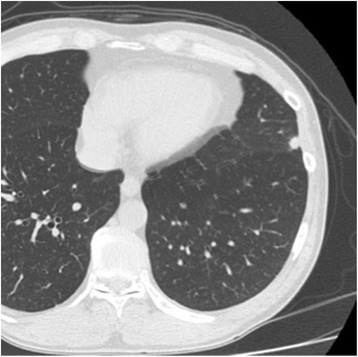
Computed tomography. A small nodule was found in the lingular segment

**Fig. 2:**
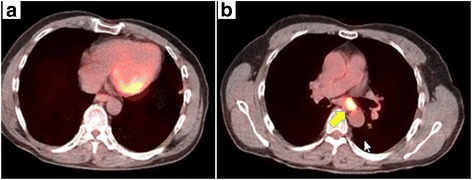
Positron emission tomography. **a**, **b** FDG uptake was detected in a subcarinal lymph node and not in a nodule in the lingular segment. The arrow indicates the lymph node with FDG accumulation

The patient was a 56-year-old man in whom a nodular shadow was found in the left lower lung field via chest radiographic screening. Because close examination via computed tomography (CT) revealed a nodular shadow in the left lingular segment, the patient was referred to our department for surgery. He was an active smoker who consumed 1 pack/day for 30 years. No other noteworthy features were found in his past history or physical findings. The CT scan showed a 1-cm nodule in the left lingular segment (Fig. 1). However, positron emission tomography revealed fluorine-18 deoxyglucose (FDG) accumulation in the subcarinal lymph node and not in the nodular shadow in the lingular segment (Fig. 2a, b). FDG uptake was not noted in other organ lesions. Serum carcinoembryonic antigen (CEA) was elevated at 14.3 mg/ml (normal range 0.0–5.0 ng/ml). Biopsy of the lesions in the lung and the lymph node was performed for a definite diagnosis.

The patient underwent thoracoscopic surgery via two incisions by video-assisted thoracoscopic approaches: a 12-mm camera port incision was made at the eighth intercostal space, the posterior axillary line; and a 4-cm incision at the fourth intercostal space, the anterior axillary line. At the time of surgery, a 1-cm nodule was palpated in the lingular segment, and wedge resection at that site was performed. Intraoperative pathologic examination revealed inflammation and necrosis. The subcarinal lymph nodes were also resected and examined pathologically. Because the results indicated a cancer lesion, the remaining subcarinal and hilar lymph nodes were dissected. The patient was diagnosed with mediastinal lymph node cancer, and no additional lung resection, such as left upper lobectomy, was performed.

**Fig. 3:**
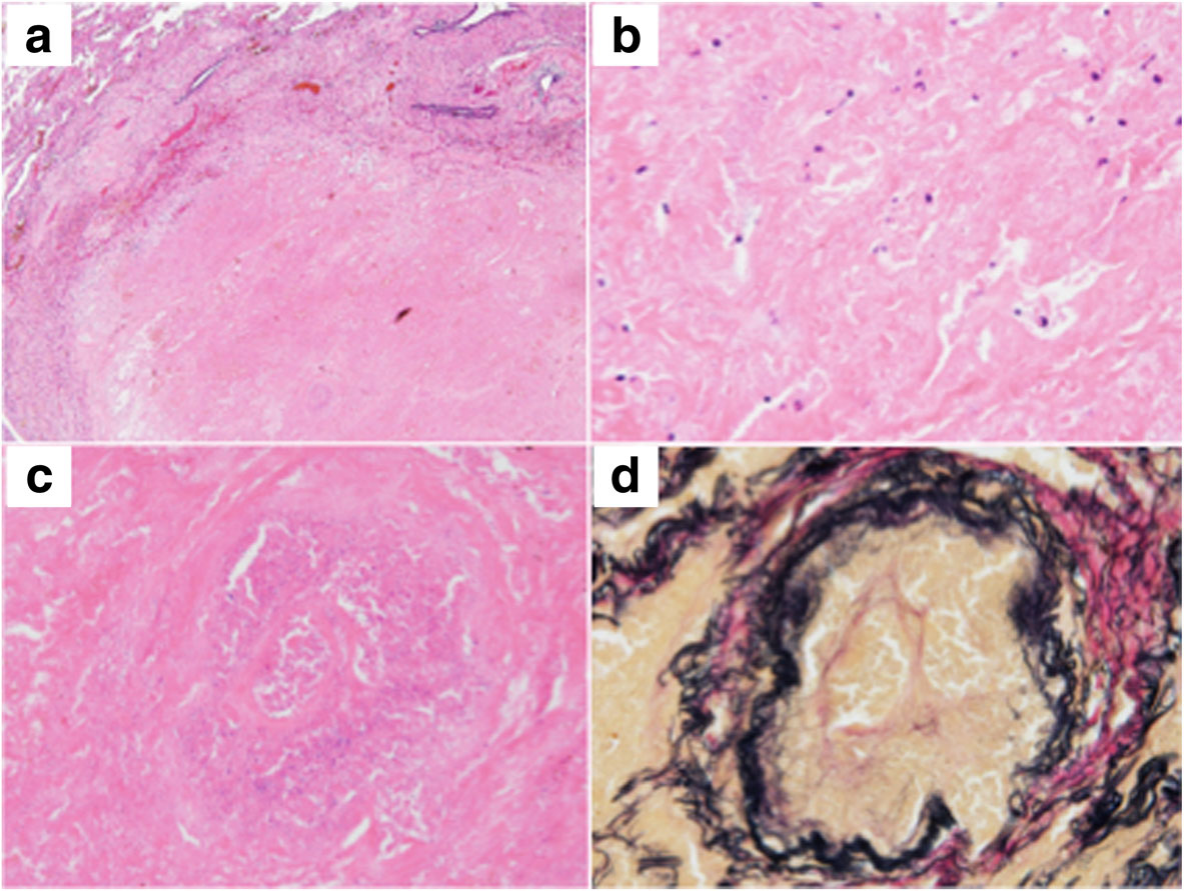
Histopathologic findings of the pulmonary nodule. **a**, **b** No viable cells could be observed inside the tumor (hematoxylin and eosin stain. **a** low magnification, **b** high magnification). **c**, **d** Vein filled with necrotic cells were observed (**c** hematoxylin and eosin stain; **d** Elastica van Gieson stain)

**Fig. 4:**
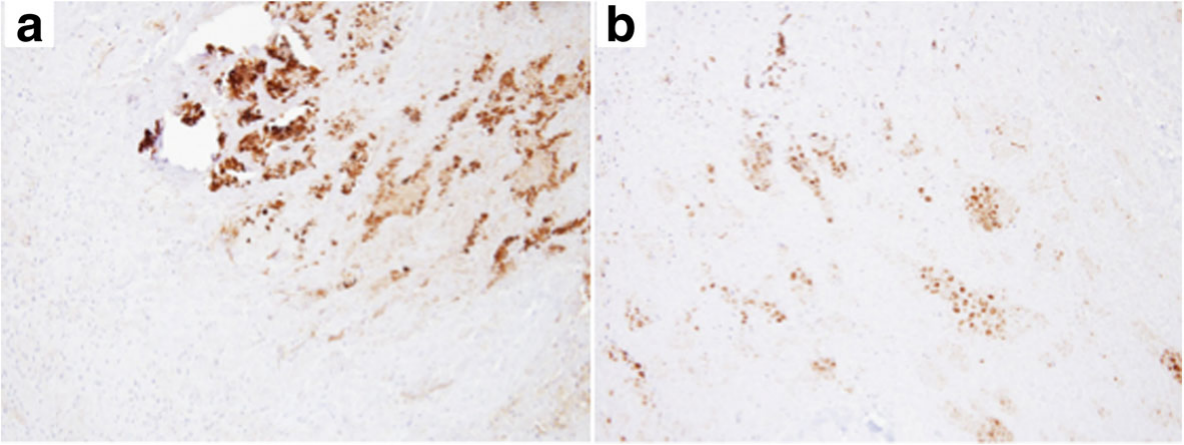
Immunohistochemical findings of the pulmonary nodule. Immunostaining results of the necrotic tissue were positive for pan-cytokeratin (AE1/AE3) and napsin A (**a** pan-cytokeratin immunostaining; **b** napsin A immunostaining)

**Fig. 5:**
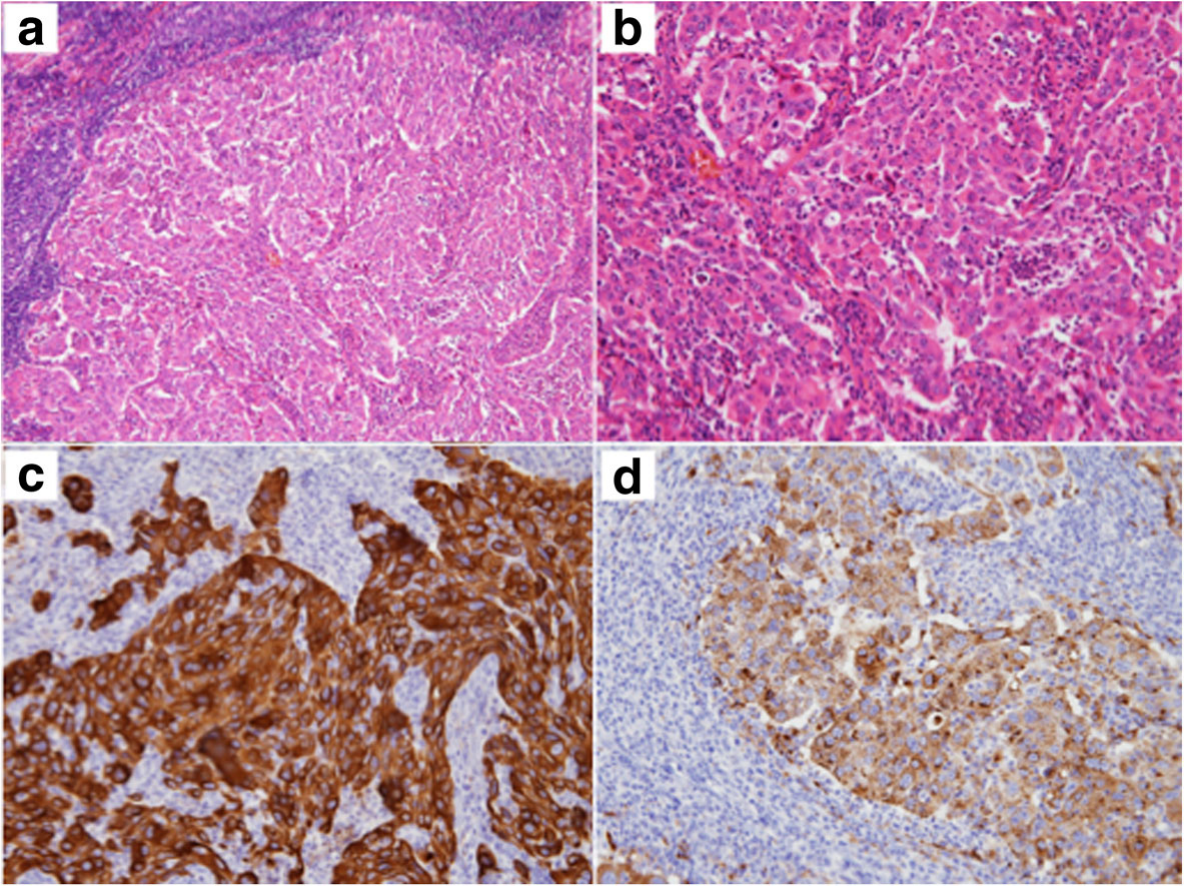
Histopathologic findings of the subcarinal lymph node. **a**, **b** Solid proliferation of cancer cells was observed (hematoxylin and eosin stain. **a** low magnification, **b** high magnification). **c**, **d** Tumor cells were positive for pan-cytokeratin and napsin A (**c** pan-cytokeratin immunostaining; **d** napsin A)

Histopathologic examination of the intrapulmonary nodule revealed necrosis and no viable tumor cells inside (Fig. 3a, b). Alveolar elastic fibers were well-maintained, and veins full of necrotic cells were observed (Fig. 3c, d). Granuloma was not evident, and acid-fast staining provided no evidence of acid-fast bacteria. Immunostaining showed irregular features positive for pan-cytokeratin (AE1/AE3) and napsin A in the necrotic tissue, suggesting residual cancer tissue (Fig. 4a, b). Meanwhile, a proliferation of large cancer cells with eosinophilic cytoplasm was found in the lymph node specimen (Fig. 5a, b). Cancer cells were found in the subcarinal lymph node, but not in additionally dissected regional lymph nodes. Immunostaining of the tumor cells was positive for pan-cytokeratin, TTF-1, and napsin A but negative for p40 and p63, suggesting a poorly differentiated adenocarcinoma of pulmonary origin (Fig. 5c, d).

**Fig. 6:**
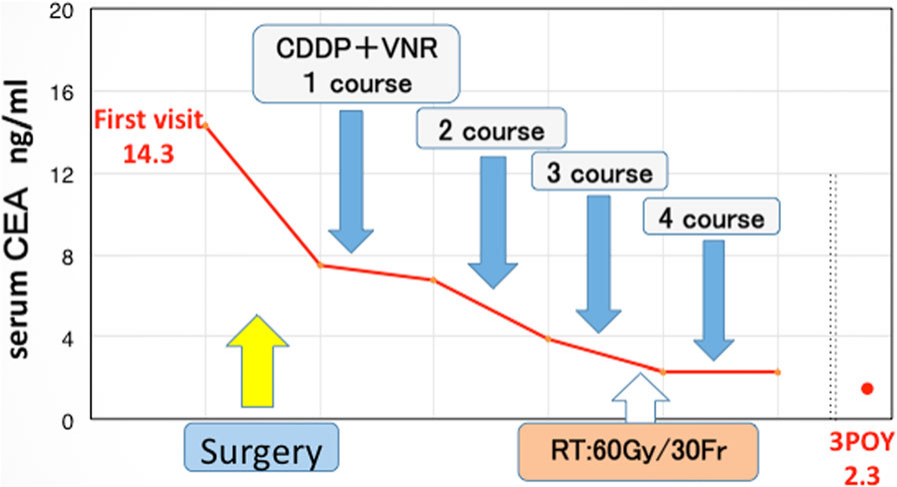
Time-course changes in the serum CEA level. The abscissa denotes time, and 1 scale corresponds to 1 month. Parallel dotted lines indicate omission of an intermediate step

The postoperative course was favorable, and the patient was discharged on the fifth postoperative day. Given that the cancer was at pTxN2M0 and stage IIIA according to lung cancer staging, concurrent chemoradiotherapy comprising systemic chemotherapy (cisplatin and vinorelbine) and radiotherapy of 60 Gy/30 Fr was administered. The serum CEA level reverted to normal after surgery (Fig. 6). To date, at 3 years after surgery, no recurrence has been noted.

## Discussion

CUP is the collective term for a group of cancers for which the anatomical site of origin remains unidentified after a metastatic focus is found [6]. CUP is characterized by clinically unconfirmed primary malignancy, early occurrence of dissemination, rapid progression, and difficult prediction of the metastatic pattern [2, 6]. The incidence of CUP is reported to be approximately 0.5–6.7% [7–10]. It occurs more frequently in men than in women, and most frequently involves the lymph nodes and bones [10]. Histologically, most cases are adenocarcinomas [10]. CUP rarely occurs in mediastinal lymph nodes, accounting for only 1.0–1.5% of all of the CUP cases [9, 10].

A possible pathogenesis of lymph node CUP is a small primary focus that cannot be detected with diagnostic imaging [3] or spontaneous resolution of the primary focus [10, 11]. Some immunologic mechanisms may be involved in the spontaneous resolution of the primary focus [11, 12]. Lymph node cancer of unknown primary origin possibly occurs if the primary focus is resolved spontaneously by host immunity, while a metastatic lymph node evades immune reactions. On the other hand, the lymph node itself may be the primary focus. In the latter case, malignant transformation of the ectopic epithelium in the lymph node may be responsible [10, 13–15]. However, all of these mechanisms are hypothetical and not based on scientific evidence.

In approximately 40% of reported cancers in the hilus, mediastinum, and cervical lymph nodes, the site of primary malignancy was the lung [10]. Taking into account the pathway of regional lymph flow in the lung, occult microcarcinoma can be considered to be present in the lung in cases of mediastinal lymph node CUP. Therefore, mediastinal lymph node cancer is commonly treated based on the assumption that the primary focus is lung cancer. In general, CUP prognosis is poor, with a median survival period of 2–9 months and a 5-year survival rate of 2.8–6.0% [2, 8]. Meanwhile, mediastinal lymph node CUP follows a clinical course different from that of CUP in general, and the prognosis is favorable when the localized focus in the lymph node is resected [3, 5, 16]. In cases of mediastinal lymph node enlargement, a surgical approach should be considered for both diagnostic and therapeutic purposes, even if the primary focus cannot be identified.

In our case, the mechanism of metastasis can be explained by lymphatic metastasis along the regional lymph flow, assuming that the primary focus was the nodule in the lingular segment and that the metastatic site was a subcarinal lymph node. The positive pan-cytokeratin and napsin A staining and the presence of necrotic cells in the blood vessels indicated the previous existence of cancer at that pulmonary site. There was no recurrence, including new primary lesions, during the 3-year follow-up after surgery noted in our patient. Thus, lymph node metastasis originating from lung cancer was highly probable based on the above reasons, although a definitive diagnosis is not possible. We believe that this case of mediastinal lymph node cancer exemplifies the hypothesis that a primary focus of lung cancer becomes necrotic through an immunologic mechanism during the clinical course, and a metastatic focus in the mediastinal lymph node alone survives and grows. Kohdono et al. reported a case of an increased metastatic focus in the mediastinal lymph node, with the primary lung cancer resolving spontaneously during the clinical course [4]. Although our present case resembles their case in some respects, to our knowledge, this is the first case of a pathologically verified etiologic mechanism of mediastinal lymph node cancer with the primary lesion remaining as necrotic tissue in the lung.

Recently published case reports regarding cancer of the intrathoracic lymph nodes were reviewed [3, 4, 16–25]. The patient characteristics of all 18 cases (including our case) are shown in Table 1. Fifteen patients were men and three were women; their average age was 63.5 years (range, 40–83 years). Except for one female patient, all patients were smokers. Seven patients had adenocarcinomas, six had squamous cell carcinomas, three had large cell carcinomas, and one had a small cell carcinoma. In 13 patients, certain tumor markers exceeded the normal range. The CUP was located in the lymph nodes of the mediastinum in 14 cases and in the pulmonary hilum in 4 cases. Surgery was performed in 13 patients, and adjuvant treatment after CUP resection consisted of radiation therapy in 4 cases, chemoradiation therapy in 3 cases, and no additional treatment in 6 cases. The outcomes after the treatment were as follows: 15 patients remained alive without recurrence or disease progression at an average of 28.8 months (range, 3–82 months), two patients remained alive with recurrence at an average of 66 months (6 and 126 months), and one patient died because of the disease 6 months after the treatment.

**Table 1:** Cancers of the intrathoracic lymph nodes with unknown primary site

Case	Author	Age (years)	Sex	Smoking history	Histology	Elevated TMs	Location	Treatment	Adjuvant treatment	Follow-up (months)	Outcome
1	Morita Y	56	Male	+	SQ	None	Med	LB and LND	None	20	Alive without recurrence
2	Kohdono S	67	Male	+	LA	None	Med	Tumor resection	RTx	6	Died of recurrence
3	Kohdono S	58	Female	+	LA	CEA	Med	LB and LND	RTx	6	Alive with recurrence
4	Kohdono S	56	Male	+	Small	SCC	rt. Hilum	LB and LND	None	16	Alive without recurrence
5	Blanco N	56	Male	+	SQ	no data	Med	Tumor resection	CRT	No data	Alive without recurrence
6	Kawasaki H	69	Male	No data	LA	CEA	rt. Hilum	LB and LND	None	20	Alive without recurrence
7	Tomita M	56	Male	+	SQ	SCC	lt. Hilum	Tumor resection	None	32	Alive without recurrence
8	Miwa K	72	Male	+	SQ	CEA	Med	LND	RTx	82	Alive without recurrence
9	Miwa K	78	Male	+	AD	CEA	Med	LND	RTx	44	Alive without recurrence
10	Miwa K	70	Male	+	SQ	None	Med	CRT	N/A	33	Alive without disease progression
11	Miwa K	76	Male	+	Undifferentiated	CEA	Med	CRT	N/A	24	Alive without disease progression
12	Shiota Y	69	Male	+	AD	CEA	Med	CRT	N/A	22	Alive without disease progression
13	Harada H	83	Female	No data	AD	NSE	Med	Tumor resection	None	38	Alive without recurrence
14	Watanabe N	55	Female	–	AD	CEA	Med	ALK-TKI	N/A	3	Alive without disease progression
15	Kim M.J.	59	Male	+	SQ	None	rt. Hilum	PN and LND	CRT	No data	Alive without recurrence
16	Kawasaki H	40	Male	+	AD	CEA	Med	LB and LND	None	126	Alive with recurrence
17	Yamasaki M	67	Male	+	AD	CEA	Med	EGFR-TKI	N/A	8	Alive without disease progression
18	Present case	56	Male	+	AD	CEA	Med	LND	CRT	32	Alive without recurrence

*TM* tumor marker, *AD* adenocarcinoma, *SQ* squamous cell carcinoma, *LA* large cell carcinoma, *Undif* undifferentiated carcinoma, *SCC* squamous cell carcinoma antigen, *CEA* carcinoembryonic antigen, *NSE* neuron-specific enolase, *Med* mediastinum, *LB* lobectomy, *LND* lymph node dissection, *PN* pneumonectomy, *CRT* chemoradiation therapy, *ALK* anaplastic lymphoma kinase, *EGFR* epidermal growth factor receptor, *TKI* tyrosine kinase inhibitor, *RTx* radiation therapy

Completely resected mediastinal lymph node CUP reportedly has a better prognosis than lung cancer with mediastinal lymph node metastasis [4, 10]. Complete resection is the first-line treatment if the lesion of the lymph node cancer of the hilum or mediastinum is localized [3, 5, 16]. Although the lung is highly likely to be the primary site of metastatic mediastinal lymph node cancer, lobectomy is generally avoided to preserve respiratory function. Even though chemoradiotherapy is often adopted as a postoperative treatment modality, there is no consensus on its therapeutic efficacy. In this case, postoperative chemoradiotherapy was performed at the request of the patient.

## Conclusion

This case shows that lymph node CUP may occur due to the regression of the carcinoma itself at the primary site. As such, aggressive treatment, including surgical resection, should be performed for mediastinal lymph node CUP.

## Abbreviation

CEA: Carcinoembryonic antigen
CT: Computed tomography
CUP: Cancer of unknown primary site
FDG: Fluorine-18 deoxyglucose
